# Computational modeling and analysis of medical resource shortages in hospital alliance: A simulation-driven approach

**DOI:** 10.1371/journal.pone.0330871

**Published:** 2025-08-26

**Authors:** Zhenkun Gan, Zhengtang Fu, Peiwu Dong, Yanbing Ju, Yajun Shen

**Affiliations:** 1 School of Economics, Beijing Institute of Technology, Beijing, China; 2 School of Environment, Tsinghua University, Beijing, China; 3 School of Management, Beijing Institute of Technology, Beijing, China; Peking University, CHINA

## Abstract

Hospital alliances, as an innovative model of hospital management, are dedicated to achieving resource sharing across the entire healthcare network. During the COVID-19 pandemic in China, these alliances played a pivotal role in combating the outbreak. However, a significant challenge emerged: the difficulty in accurately quantifying medical resource shortages at individual hospitals hindered the efficient allocation of these critical resources. To address this issue, this study proposes an integrated urgent medical resource evaluation model designed to scientifically assess the urgency of medical resource needs within the alliance. Methodologically, the model innovatively combines the SEIR system dynamics model, complex network analysis, and entropy-weighted TOPSIS to construct a multi-dimensional evaluation framework. A case study has been conducted to validate the effectiveness of the proposed methodology. Contrary to conventional expectations, the findings reveal that small-scale hospitals exhibit higher medical resource urgency compared to their large-scale counterparts within the alliance. Based on these results, we recommend that policymakers prioritize addressing medical resource shortages in small-scale hospitals during pandemics.

## 1. Introduction

During public health emergencies, people urgently seek medical treatment and purchase medicines to alleviate the physical discomfort and psychological stress caused by illness. However, the rational allocation of medical resources during epidemics remains a persistent and significant challenge for hospitals [1,2]. During the SARS and the COVID-19 epidemic in China, the problem of irrational allocation of emergency medical resources was very urgent, which directly affected the treatment process of patients. During the Guangdong SARS epidemic in 2003, the distribution of medical resources among hospitals at all levels was irrational, in which the small-scale hospitals (e.g., community hospitals) had a large number of patients, but the medical resources were relatively scarce, and large number of medical resources were allocated to the large-scale hospitals, which resulted in the inability to meet the demand of patients in small hospitals [3,4]. In public health emergencies, proper management of emergency medical allocation is a key issue in China’s healthcare reform and also is a hot issue related to people’s interests in healthcare.

**Fig 1:**
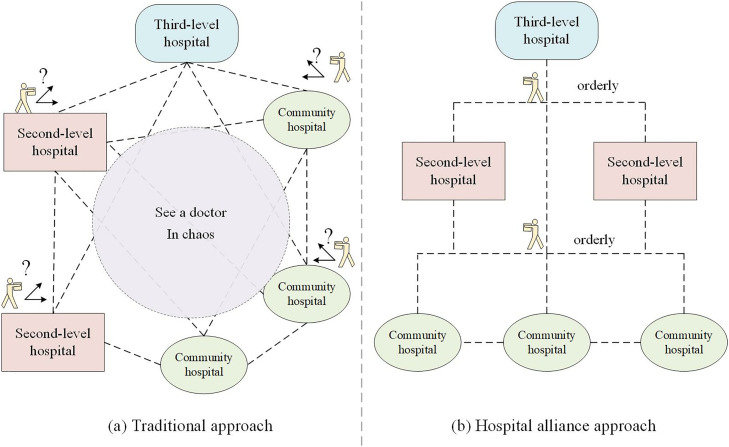
The comparison of medical resource allocation.

To address the challenge of medical resource allocation during public health emergencies, China has implemented the innovative operational mechanism of hospital alliances, which has demonstrated effective results in optimizing resource distribution. [5]. As shown in Fig 1, compared with the traditional medical care process, the use of the hospital alliance method of drug distribution can facilitate the consultation and drug collection of the medical masses through the orderly referral mechanism, avoiding the people’s blind consultation and drug collection. For example, during the Beijing Omicron epidemic at the end of 2022, Beijing chaoyang district hospital alliance (China-Japan friendship hospital alliance) relied on the hospital alliance operational mechanism to optimize the distribution of medical resources, which achieved great efficiency [6]. During public health emergencies, we could help patients obtain medical treatment in order based on the mechanism of the hospital alliance. In this way, the hospital alliance could optimize the allocation process of medical resources, and improve the patient’s medical treatment feelings.

Supported by the operational mechanism of hospital alliances, hospitals can achieve the goal of integrated medical resource management [7]. For the distribution of medical supplies in the alliance, all member hospitals use a unified drug list catalog and share the same inventory management information system, thus realizing the unified distribution and management of supplies within the alliance. Especially in the case of public health emergencies, the alliance can carry out an orderly flow of medicines and devices among member hospitals without worrying about the poor circulation of materials within the alliance due to different specifications and categories. The lead hospital of the alliance can undertake the duty of procurement and scientific distribution of medical resources. For example, during the Omicron COVID-19 epidemic, Shenzhen Luohu hospital alliance adopted this mechanism for medical resource management and achieved great results. Luohu People’s Hospital undertook the function of distributing medical resources to member hospitals and strongly guaranteed the orderly diagnosis and treatment of the whole hospital alliance. However, the hospital alliance is an emerging hospital organization pattern, that still needs to be improved, especially in the quantification of medical resources’ urgency.

This paper proposes a novel medical resource evaluation model for hospital alliances to address the lack of urgency quantification methodology. The contributions are threefold.

(1) This paper proposed the concept of medical resource urgency degree to characterize the severity of resource shortages during pandemics.
(2) This paper proposed a systematic evaluation index system for medical resources urgency, especially in major public health emergencies.
(3) This paper introduced an integrated evaluation model with system dynamics and the TOPSIS model to quantify the resource urgency degree of individual hospitals.

The remainder of this paper is arranged as follows: **Section** **2** reviews the related work and proposes the research gaps. **Section** **3** proposes the hospital alliance shortage degree evaluation framework. **Section 4** puts forward the mixed urgency evaluation model to analyze the performance of this sharing system. **Section** **5** shows the case study of the Beijing Haidian hospital alliance in the context of the pandemic. **Section** **6** summarizes this work and proposes future research directions.

## 2. Literature review

### 2.1 Resource allocation and management

The resource allocation problem has attracted much attention both in the ordinary context and emergency context, as shown in Table 1. Rubio et al. (2020) studied the shortage of protective masks during the new coronavirus pneumonia epidemic, and by grading different mask protection types and capabilities, sorted out the types of masks with serious shortage risks, providing new ideas for the management of multicategory emergency supplies [20]. Kumar et al. (2021) conducted an in-depth study and risk modeling for the large-scale food supply chain shortage problem under the scenario of a new crown pneumonia epidemic. Based on a fuzzy max-min decision model, the uncertainty of the occurrence of the epidemic was mathematically described and combined with a supply chain model. Based on the simulation and analysis, it was concluded that the supply chain disruption due to the epidemic can be effectively mitigated by using this emergency decision model [21]. EI et al. (2021) investigated the supply chain risk management problem during the New Crown Pneumonia epidemic, and based on the empirical research, it was concluded that the information processing capability plays an important role in reducing the supply chain risk during the emergency scenario by collecting the data of 470 supply chain companies. It can enhance the resilience and robustness of the supply chain [22]. Nagurney et al. (2021) studied the shortage of material supply chain under the new crown pneumonia epidemic from the perspective of game theory and analyzed the mechanism of the labor shortage factor’s role and the degree of influence on the emergency supply chain by constructing a supply chain network game model [23]. Mbaigoto et al. (2025) focus on the water resource management model and proposed a new construct to analysis the social impaction on the resource management [24].

**Table 1:** Summary of literature for resource management.

Research topic	Methodology	Reference
Resource synergistic allocation for multi-locations	Stochastic programming	Doan et al. (2019) [8]
Resource synergistic allocation	Multi-layer network optimization model	Pradhananga et al. (2016) [9]
Resource allocation in post-disaster	Mixed integer programming and meta-heuristics algorithm	Jain et al. (2022) [10]
Resource dynamic programming in the initial stage	Dynamic programming	Friedrich et al. (2000) [11]
Emergency resource allocation of humanitarian aid	Multi-objective optimization an dmulti-attribute decision-making	Sarma et al. (2019) [12]
Emergency medical resource allocation	System simulation	Dees et al. (2023) [13]
Emergency equipment allocation	Data mining and Maximum set coverage	Zonouzi et al. (2020) [14]
Emergency resource allocation	Machine learning and regression analysis	Lee et al. (2020) [15]
Emergency resource dynamic allocation	multi-objective optimization	Zhang et al. (2020) [ 16]
Medical resource evaluation	Improved ORESTE method	Gou et al. (2024) [17]
Resource evaluation model	Fuzzy multicriteria evaluation model	Skare et al. (2023) [18]
Natural resource evaluation	Data-driven approach	Lyra et al. (2023) [19]

### 2.2 Hospital alliance management

The research work on the management of hospital alliances is in the initial stage, mainly focusing on the two aspects of the management of the operational efficiency of hospital alliances and the safe sharing of data on the operation of hospital alliances. In the research on the operational efficiency management of healthcare alliance, Andrews et al. (2020) studied the flow model of healthcare personnel in a community clinic alliance, and based on the idea of optimization design of engineering process, carried out process reengineering of human resource allocation and scheduling of healthcare personnel in clinic alliance [25]. Sparks et al. (2021) studied the service effectiveness of specialty hospital alliances under the new crown epidemic and conducted an empirical study through actual diagnosis and treatment data, and the results of the study showed that specialty hospital alliances have better diagnosis and treatment service resilience under the new crown epidemic model, which plays an important role in improving the satisfaction of the visiting population [26]. Lavi-Rotenberg et al. (2020) studied the organization of the psychological diagnosis and treatment hospital alliance and the problem of patients’ consultation and verified that the alliance mechanism has a better effect on the psychological diagnosis and treatment service in the psychological diagnosis and treatment service [27].

Synergistic analysis of hospital alliances is also a popular research topic. Wiggins et al. (2021) investigated the effective synergistic effects of the alliance model of psychotherapy with multisectoral participation. They used a combination of literature research and qualitative research to evaluate the synergistic effect of hospital alliances under multisectoral participation [28]. Cossio-Gil et al. (2022) studied the operation and management of hospital alliances in Europe, and based on the empirical analysis methodology, they proposed that adopting the hospital alliance model can improve the mutual satisfaction of patients, hospital administrators, and medical equipment service providers, with a distinct synergistic and mutually beneficial effect [29]. Verhulst et al. (2013) studied the effectiveness of healthcare consortia in improving the diagnostic and treatment capacity of healthcare organizations, and based on the comparative experimental method, it was proved that the use of healthcare consortia can improve the efficiency of doctors’ diagnosis and treatment and the patient’s satisfaction [30]. Puro et al. (2024) focus on the hospital partnership evaluation and proposed the data-driven approach to quantify the collaboration preference with The American Hospital Association Annual Survey provided data [31].

In terms of secure data sharing for hospital alliance, Dhiman et al. (2022) studied the privacy management of healthcare data in hospital alliances and proposed to adopt the Byzantine Consensus healthcare data sharing platform for collaborative management of hospital alliance data, thus facilitating patients’ access to different hospitals [32]. Mohiyuddin et al. (2022) studied the sharing and storage issues of data collected by IoT devices in hospital alliances and proposed to adopt cloud computing storage for storing and securely sharing patients’ physical function data [33]. Lipworth et al. (2022) studied the problem of sharing and storing data collected by IoT devices in hospital alliances and proposed the use of cloud computing storage for storing and securely sharing patients’ bodily function data in hospital alliances [34]. Berkowitz et al. (2017) studied the security management problem of diagnosis and treatment data within the Hopkins hospital alliance in the United States. Based on the empirical analysis method, they studied the implementation path of secure sharing of medical data under the hospital alliance and demonstrated that secure sharing of patient diagnosis and treatment data can facilitate patients’ medical referrals and improve the trustworthiness and acceptance of medical data of referred patients [35]. Yaqoob et al. (2021) studied the issue of the opportunities and challenges of the secure sharing of medical and healthcare data and proposed the adoption of blockchain technology as an important support tool for the secure sharing of medical and healthcare data in hospital alliances, which is an important support tool for the secure sharing of medical data in hospital alliances [36].

### 2.3 Shortage evaluation methodology

Shortage evaluation models and methodology are critical for managers to scientifically know the scarce situations of resources. Plenty of scholars and scientists have made great efforts to enrich the methodology and knowledge base in the field. Huang et al. (2022) proposed an integrated evaluation model supported by the grey TOPSIS method and data-driven approach [37]. Haugstetter et al. (2022) proposed a novel shortage evaluation model for the Cancer Urgent Assessment Clinic to sequence the urgency of the patient in the COVID-19 context [38]. Devaraj et al. (2020) designed a new shortage evaluation framework to quickly respond to emergency needs with a deep learning model and social network analysis method [39]. Zhao et al. (2022) proposed an advanced shortage evaluation method for medical equipment logistics schedules supported by the CRITIC method [40]. Sonnenschein et al. (2021) adopted the artificial intelligence method to evaluate the shortage of peripheral vascular intervention in the clinical scenario with a classification model and random forest [41]. Chen et al. (2021) adopted the uncertainty models to evaluate equitable infrastructure restoration in emergency scenarios, and the case study shows that the proposed uncertainty model has great efficiency in the real scenario [42]. Zhang et al. (2023) designed the state-of-the-art shortage evaluation model to support the medical logistics in emergency medical scenarios with the entropy weight method, which adopted the typical indicators [43]. Altinoz et al. (2023) introduced the urgency level evaluation operator to optimize the vehicle schedule problem in the urgency period. Via the real case study, the optimization models with shortage have been verified with the reality data set [44]. Knezevic et al. (2022) proposed a novel evaluation framework to measure the shortage of vaccines in the pandemic scenario supported by real data [45].

### 2.4 Research gap

Based on the aforementioned literature review, it has been found that current research primarily focuses on hospital alliance management or resource allocation, while significant gaps remain in quantifying the urgency of medical emergency supplies within the hospital alliance context. Although hospital alliance administrators place great emphasis on assessing supply urgency across member hospitals, existing urgency calculation methods remain overly simplistic, lacking scientific rigor and systematicity—particularly in terms of quantitative models and methodologies that require further refinement. Given the notable theoretical deficiencies in current research on urgency quantification models for medical consortia, theoretical studies on this topic hold substantial academic and practical value. Therefore, systematic and in-depth exploration by the academic community is imperative.

## 3. Proposed evaluation framework

### 3.1 Resource urgency definition

Considering that the study of the urgency of the hospital alliance’s resources belongs to a relatively new research object, the academic community has not yet formed a unified standard for the connotation of the concept. This paper defines the concept of hospital alliance material urgency as follows: hospital alliance material urgency quantifies hospitals’ material demand intensity on a [0,1] scale. For example, if the resource shortage of a healthcare organization is higher, it means that the degree of the organization’s desire for material demand is also higher, and the negative losses caused by material shortages are also higher; on the contrary, if the material shortage of a healthcare organization is lower, the negative losses caused by material shortages of the node hospital are also smaller. In the calculation of the shortage of supplies in the hospital alliance, the decision-making body is usually the expert group of the healthcare hospital alliance, and the members of the expert group usually have rich experience in healthcare and can quantify the shortage degree according to the differences in the attributes of the node hospitals of the hospital alliance. In the assessment process, the expert group needs to ensure that the data is true, the calculation logic is simple, and it is easy to be accepted and understood by hospitals and patients.

Based on the above conceptual definition, this paper further defines the value range of the shortage of the hospital alliance $C_{i}$ as follows: Let the set of all member hospitals of the hospital alliance be N, the numerical results of the shortage of the hospital alliance are related to a variety of influencing factors, and the implicit function of the shortage degree $C_{i}$ is expressed as shown in Eq. (1).


$$C_{i}=f\left(k_{1},\;k_{2},\ldots ,k_{m}\right),\;i\;\in \;N$$ (1)

In this equation, the results of the resource shortage degree for any member of the hospital alliance must be satisfied $0\leq C_{i}\leq 1,\;i\in N.$

### 3.2 Shortage-related factors designing

**Fig 2:**
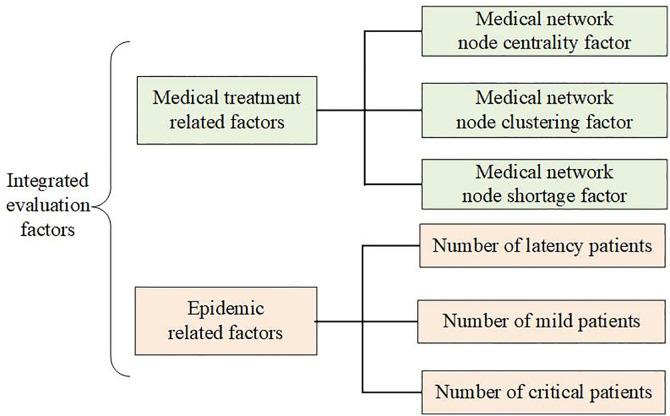
Framework of evaluation factors.

In the context of public health emergencies, the shortage of hospital alliance materials is mainly affected by two main types of factors: (1) The shortage degree of hospitals; (2)The urgency degree of disease. Among them, the diagnosis and treatment network refers to the patient referral and communication network within the hospital alliance, in the face of public health events such as epidemic outbreaks, the node members of the diagnosis and treatment network bear enormous diagnosis and treatment pressure, and there are differences in the attributes, functions, and tasks undertaken by the medical nodes at all levels, which need to be analyzed systematically; the urgency of the spread of epidemics in the hospital alliance is associated with epidemic propagation characteristics, and highly infectious diseases will lead to a surge in the number of patients and a simultaneous increase in the consumption of healthcare alliance materials, thus exacerbating the shortage of materials. This paper explores material shortages in healthcare alliances during public health emergencies, drawing on emergency logistics resource classification and National Healthcare Commission guidelines (Fig 2).

#### (1) Medical network node centrality factor.

In the hospital alliance diagnosis and treatment network, the number of connected edges of a node hospital represents the path of interaction relationship between that hospital and other hospitals in the alliance, which is expressed in terms of the degree of the node. If the number of its connecting edges is larger, it indicates that the hospital’s diagnostic and treatment role within the hospital alliance is larger, and other hospitals are more dependent on the hospital. For hospitals with a larger degree of node centrality, their consumption of supplies is also larger. In the event of a shortage of medical supplies, the node will also have a greater impact on the healthcare network of the hospital alliance. Therefore, hospitals with larger node centrality also have a higher degree of material urgency.

#### (2) Medical network node clustering factor.

The degree of aggregation of network nodes is an indicator of the association in complex networks. In the healthcare network, the following phenomenon often exists: large medical institutions and several secondary hospitals, community hospitals to form a diagnosis and treatment network, the relationship between members of different hospitals in the network is high, the frequency of patient referrals is also high, that is, with large hospitals connected to the medical institutions, there is a greater probability of the existence of neighboring relationships. Therefore, for hospitals with a high degree of node aggregation, the network impact caused by the occurrence of material shortages is also larger, and its importance and urgency are correspondingly higher.

#### (3) Medical network node shortage factor.

The medical supply gap rate is a ratio that quantifies the difference between the quantity of supplies needed by a nodal hospital and its current stock. If the material gap rate of the node hospital of the medical association is larger, it means that the material shortage of the hospital is more serious, which will have a direct impact on the diagnosis and treatment effect of the hospital. Therefore, the material gap rate element has a very important role in the urgency of the diagnosis and treatment network.

#### (4) Number of latency patients.

Latent patients are those who have been infected with the virus, but currently have no obvious symptoms, and will be converted to confirmed patients after a certain period. These patients usually do not seek medical attention because they have no obvious symptoms, and are difficult to detect with nucleic acid testing in the early stages. These patients are potentially contagious, and if the number of latent patients increases in a hospital’s jurisdiction, it will pose a greater risk to the management of the outbreak in the jurisdiction. However, since these patients do not have obvious symptoms, they do not usually seek medical attention in hospitals and the demand for medical resources is weak.

#### (5) Number of mild patients.

Confirmed mildly ill patients are those who have been infected with the virus and have mild symptoms, and these patients are generally transformed from patients in the incubation period and have a strong capacity for viral infection. In terms of demand for diagnostic and treatment resources, these patients have a moderate demand for medical supplies. For example, mildly ill patients are prone to fever, cough, and other symptoms, if the symptoms are mild can be self-limiting repair through the body’s self-healing function, the demand for medicines is not as strong as that of severely ill patients. However, during the spread of public health events, the number of patients in this category is generally higher, and the number will be higher than the number of patients with severe diseases. Therefore, the number of mildly ill patients affects the urgency of the spread of the epidemic in the hospital area and is an important factor in measuring the extent of the spread of infectious diseases.

#### (6) Number of critical patients.

Serious patients are infected people with severe symptoms that require treatment such as hospitalization. These patients are usually transformed from patients with mild illnesses and have a strong capacity for infection. In terms of the demand for diagnosis and treatment resources, seriously ill patients have a higher demand for medical resources and a higher demand for medicines due to their severe conditions. Under the hierarchical diagnosis and treatment model of the hospital alliance, critically ill patients are generally upwardly transferred from community hospitals to the lead hospitals of the hospital alliance for centralized treatment. Therefore, the number of critically ill patients affects the urgency of the epidemic situation in the hospital area and is an important factor in measuring the urgency of the spread of public health emergencies.

### 3.3 Framework of integrated evaluating model

**Fig 3:**
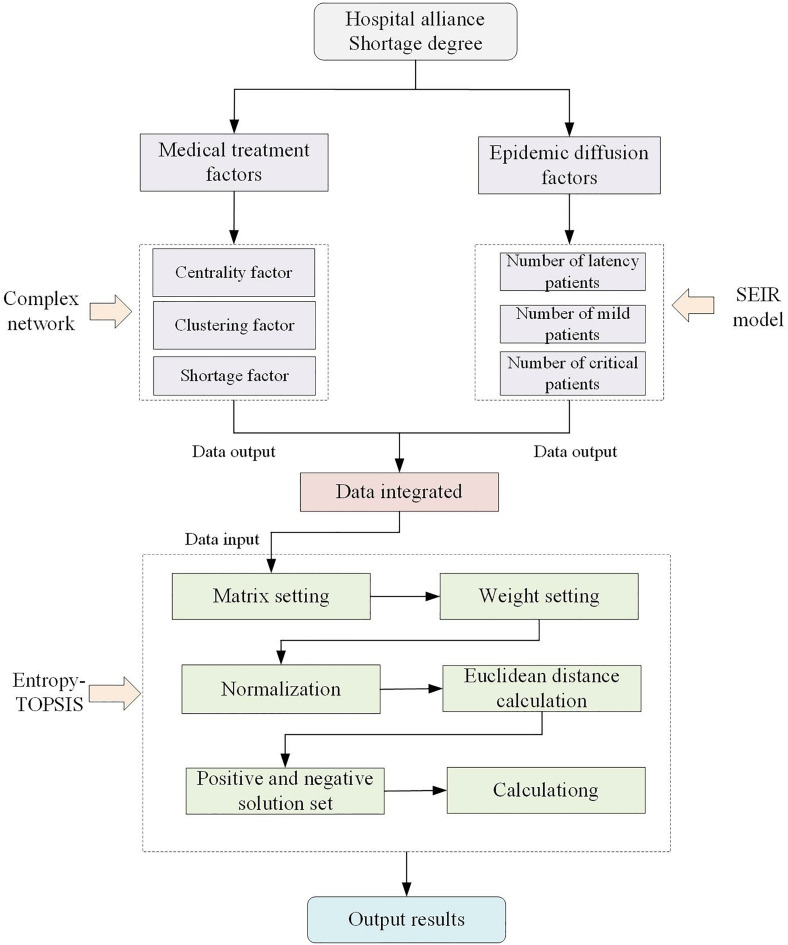
Urgency degree evaluation framework.

The process of calculating the urgency of the material of the medical association has complexity and multiplicity. Therefore, this section proposed the integrated framework to combines the complex network model, SEIR infectious disease model, and the improved entropy weight TOPSIS model to calculating the urgency of the material for the medical association. The specific calculation process can be divided into two modules are shown in Fig 3. Among them, the medical treatment-related factors will be calculated by the complex network model, while the epidemic spread-related factors will be computed using the SEIR model. The above calculated result would frame a mixed dataset and will then be further integrated, with the integration method being based on the Entropy-TOPSIS model. Ultimately, this computational process will yield urgency level values for each member institution within the hospital alliance.

In the first calculation part, the urgency impact factor values need to be quantified. In the specific calculation, the complex network model and the SEIR infectious disease model are used to calculate the medical urgency impact factor and the epidemic spread urgency impact factor of the hospital alliance, and record the calculated urgency value results. Among them, the complex network model is mainly used to calculate the three impact factors of medical network node centrality, medical network node aggregation, and medical network node material gap rate of the hospital alliance; the SEIR infectious disease model is mainly used to simulate the patient data covered by hospitals in each node of the hospital alliance, including the number of patients with the latent period, the number of patients with mild illnesses, and the number of patients with critical illnesses of the three impact factors.

In the second calculation part, the numerical results of the above urgency impact factors need to be integrated to output the quantified values of material urgency for each hospital in the hospital alliance. In this part, the entropy-TOPSIS model is adopted for the integration of the impact factors. First of all, it is necessary to determine the calculation weights of the urgency impact factors, and in the process of determining the weights. Besides, it is also necessary to ensure the scientific and reasonable calculation of the weights. After completing the determination of weights, we should calculate the Euclidean distance between each hospital and the positive and negative ideal points and calculate the final urgency value results through this distance. What should be noticed is that the weight assignment would be driven by expert experience and the consistency check would based on the critical values of the corresponding matrices.

## 4. The methodology of the integrated evaluation model

**Table:** 

Nomenclature			
i	Index of hospital	$T_{1}$	the latent period
m	Index of factors	$T_{2}$	treatment cycle
N	Set of hospitals	$\begin{array}{c} I^{\prime } \end{array}$	The number of clinically ill patients
Ω	Set of weights	$\zeta_{1}$	proportion of mild patients referred to tertiary hospitals
$k_{i}$	Degree value of the node hospitals	$\zeta_{2}$	The proportion of mild patients referred to secondary hospitals
CO(i)	aggregation value of hospital i	$\zeta_{3}$	The proportion of severe patients referred to tertiary hospitals
$E_{i}$	the number of edges of hospital i	$P_{1}$	the number of latent patients
$R_{i}$	The quantity of demanded of hospital i	$P_{2}$	the number of mild patients
$p_{i}$	The existing stock of hospital i	$P_{3}$	the number of severe patients
Q(i)	the result of the material gap of hospital i	$\overset{\wedge }{X}$	the weighted normative matrix
α	infected probability		

### 4.1 Model assumptions

The model is constructed based on the following assumptions:

(1) The disease course in affected populations comprises four phases: the latent stage, mild stage, critical stage, and eventual outcome (recovery or death).
(2) The mortality or recovery would not affect the future medical resources due to the immunity.
(3) This paper focuses on the assessment of emergency material urgency levels within the context of the medical alliance. Therefore, constraints such as transportation delays in supply chain scenarios are not considered.

The above hypothetical conditions are modeled at the macro level for outbreak infections that fit the classical infectious disease profile. There may be variability in the spread of outbreaks for different infection characteristics. Therefore, the model is applied to general scenarios and pays more attention to the ideas and general laws of model construction, thus providing scientific model support for global infectious disease emergency supply allocation.

### 4.2 Complex network model

The hospital alliance is a hospital network system, which has nodes (hospitals) and linkages (treatment transfer relationships). Hence, the measurement of the medical emergency should also consider the medical network features. Three factors should be quantified in the evaluation model.

#### (1) Calculation of node centrality.

In the diagnosis and treatment network, the number of edges of the node hospital represents the number of referral contacts between the hospital and other hospitals, using the degree value. In the diagnosis and treatment network, a node with a higher degree value indicates that the hospital holds a more important position in the network and plays a greater role in supporting the medical system’s operations. However, relying only on degree value to measure node attributes in the diagnosis and treatment network is insufficient, as it fails to fully reflect the node’s overall importance within the network.

Node centrality is a measure of node connectivity attributes from the perspective of the network as a whole, which is expressed as the proportion of the node’s degree value to the cumulative degree value of all the nodes, reflecting the status of the point in all the nodes, and is more comprehensive and representative. The larger the node centrality degree of the diagnosis and treatment network, the more important the node is, and the specific calculation process is shown in Eq. (2).


$$D\left(i\right)=\frac{k_{i}}{\sum {\mathrm{\backslash nolimits}}_{i=1}^{N}k_{i}},$$ (2)

Where $k_{i}$ is the degree value of the node hospitals, representing the number of hospitals that have direct referral links with the nodes. $\sum {\mathrm{\backslash nolimits}}_{i=1}^{N}k_{i}$ denotes the cumulative sum of the degree values of all node hospitals in the healthcare network.

#### (2) Medical network clustering factor.

In the healthcare network, there is a tendency for different hospitals to aggregate in groups, presenting the characteristics of medical network grouping, i.e., there is a high probability for different hospitals to establish referral links through the same hospital, e.g., if a tertiary hospital has a referral link with a secondary hospital, and that secondary hospital has a referral link with a certain community hospital, the community hospital, and that tertiary hospital has the probability of referral to each other as well.

In complex network modeling systems, network node aggregationCO(i) is used to depict this aggregation and bridging relationship. The larger the node hospital, the more connected the hospitals adjacent to the node hospital are likely to be. The aggregation is calculated as shown in Eq. (3).


$$CO\left(i\right)=\frac{2E_{i}}{k_{i}\left(k_{i}-1\right)},$$ (3)

The above equation $k_{i}$ denotes the degree of the node hospital, $E_{i}$ denotes the number of edges that exist between the node hospitals directly neighboring the node hospital, and $k_{i}(k_{i}-1)$ denotes the maximum number of edges that can exist in the diagnosis and treatment network.

#### (3) Medical network resource gap ratio.

The resource gap ratio represents the degree of material unmet in a nodal hospital and plays an important role in the effectiveness of hospital treatment. In the calculation of the material gap rate, the difference between the material demand and the material stock can be used to calculate the unsatisfied amount of the material in each hospital, and then the material gap ratio can be deduced from the unsatisfied amount. The method of calculating the material gap degree of each member hospital of the medical association is shown in Eq. (4)$R_{i}$ denotes the quantity of materials demanded by the node hospital, $p_{i}$ denotes the existing stock of materials in the node hospital, and Q(i) denotes the result of calculating the material gap of each node hospital.


$$Q\left(i\right)=\frac{R_{i}-p_{i}}{R_{i}},$$ (4)

### 4.3 SEIR system dynamics model designed

This paper considered the basic law of patients from virus infection to disease diagnosis and combined it with the characteristics of epidemic transmission in the jurisdiction of each hospital. The SEIR model is set up as follows: (1) The latently infected person (E)is also set up as a patient with the ability to transmit the virus, and (2) In the above models, some of the patients will be treated at home. Therefore, the concept of home isolation rate is introduced in the model construction, which makes the model more consistent with the epidemic transmission law under the mechanism of medical association.

**Fig 4:**
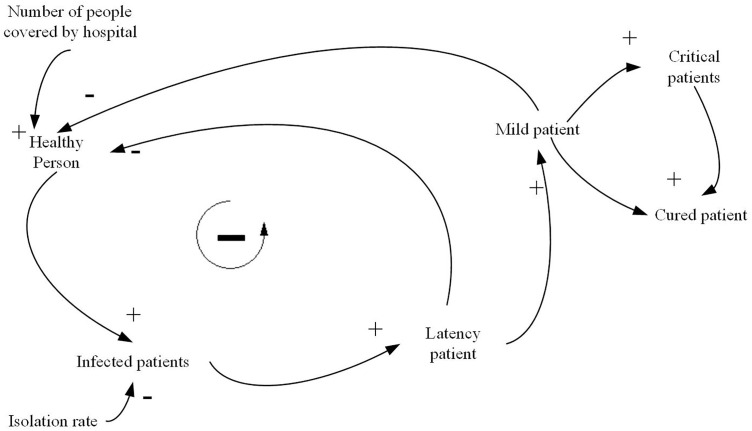
SEIR causal loop diagram.

The causal loop relationship of the model is shown in Fig 4. There is a negative feedback loop in the causal loop diagram: susceptible population → number of latent daily viral infections → latent patients → clinically diagnosed patients → susceptible population. Considering that the negative feedback loop has the characteristic of cyclic regulation, it is necessary to use simulation to deduce and analyze the relevant variables involved in the loop in the modeling and determine the final results of each variable.

The differential equation for the spread of the epidemic is shown as follows.


$$\begin{array}{c} \frac{dS^{\prime }}{dt}=-S^{\prime }\alpha \beta \frac{I(1-\theta )\left(1+\sigma \right)}{N}, \end{array}$$ (5)

$$\begin{array}{c} \frac{dE^{\prime }}{dt}=S^{\prime }\alpha \beta \frac{I(1-\theta )\left(1+\sigma \right)}{N}-\frac{E^{\prime }}{T_{1}}, \end{array}$$ (6)

$$\begin{array}{c} \frac{dI^{\prime }}{dt}=\frac{E^{\prime }}{T_{1}}-\frac{I^{\prime }}{T_{2}}, \end{array}$$ (7)

Eq.(5) describes the process by which a susceptible person is infected by the virus. The infectious velocity (Missing dimension or its units for \kern dS/dSdt\nulldelimiterspacedt) is related to the Number of people in the hospital district ($\begin{array}{c} S^{\prime } \end{array}$), infected probability(α), elder rate, Isolation rate, and connected people ($\beta \frac{I(1-\theta )}{N}$). Due to the viral infection reducing the number of susceptible individuals, the equation has a negative correlation. Eq. (6) describes the process of change in the number of latent patients, the increase in the number of latent patients comes from the infection of susceptible persons, the rate of increase is (−dS′/dS′dt\nulldelimiterspacedt), and patients in the latent period, after the onset of the disease, will be converted to clinically ill patients ($\begin{array}{c} I^{\prime } \end{array}$), and the rate of conversion is (−E′/E′T1\nulldelimiterspaceT1), which is negatively correlated, because of the existence of the latent period ($T_{1}$). Eq. (7) describes the change in the number of patients with clinical morbidity ($\begin{array}{c} I^{\prime } \end{array}$), with a positive correlation between the rate of conversion of patients in the latent phase to patients with clinical morbidity (E′/E′T1\nulldelimiterspaceT1), and the conversion of patients with morbidity to patients who recover or die after receiving clinical treatment after a certain treatment cycle ($T_{2}$).

**Fig 5:**
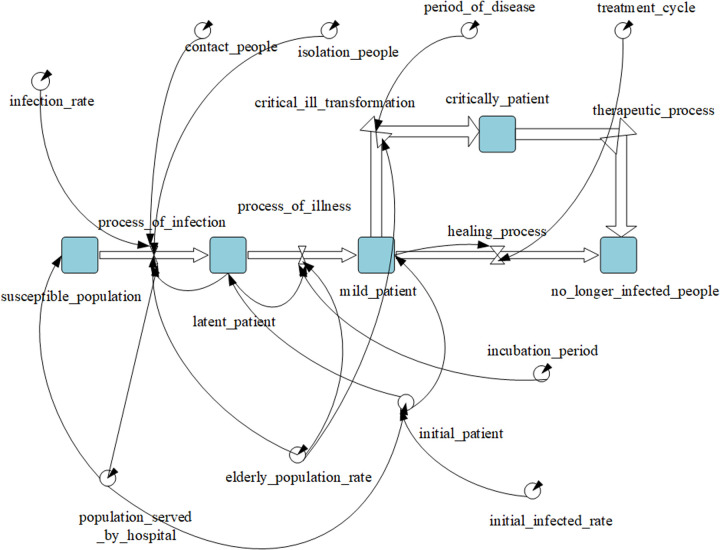
SEIR System dynamics model.

After completing the construction of mathematical relationships between elements, the model can be constructed in the simulation environment. This paper adopts Anylogic to run the system dynamics model. In the simulation, the stock variables are the number of susceptible persons, the number of persons in incubation, the number of persons diagnosed, and the number of persons no longer infected. The flow variables are the infection process, the disease onset process, and the cure process, and the rest of the variables are system input constants, including the resident population in the jurisdiction of the hospital alliance, the infection rate, the isolation rate, the average number of people exposed to the virus, the viral incubation period, and the treatment period, and the specific system dynamics model is shown in Fig 5.

After completing the above simulation, the patient can be referred based on the referral characteristics of the hospital alliance to achieve the hierarchical diagnosis and treatment goal. This ensures that most patients with mild conditions are treated in community hospitals, a small number of mild patients are referred to secondary or tertiary hospitals, and very few patients (severe cases) are transferred to tertiary hospitals.

During actual referrals, the number of mild and severe patients in each hospital will change, while the number of latent patients remains unchanged. In the calculation process, let: The proportion of mild patients referred to tertiary hospitals is $\zeta_{1}$. The proportion of mild patients referred to secondary hospitals is $\zeta_{2}$. The proportion of severe patients referred to tertiary hospitals is $\zeta_{3}$.

During the simulation, the scale of latent patients $P_{1}$, mild patients $P_{2}$, and severe patients $P_{3}$ in each hospital can be directly read from the simulation software panel. After implementing the referral in the hospital alliance, the scale of latent patients $P_{1}^{*}$, mild patients $P_{2}^{*}$, and severe patients $P_{3}^{*}$ in each hospital needs to be recalculated as shown in Eq. (8)–Eq. (10).


$$P_{1}^{*}=P_{1}$$ (8)

$$P_{2}^{*}=P_{2}-P_{2}\times \zeta_{1}-P_{2}\times \zeta_{2}$$ (9)

$$P_{3}^{*}=P_{3}-P_{3}\times \zeta_{3}$$ (10)

### 4.4 The improved entropy-TOPSIS model

The TOPSIS (Technique for Order Preference by Similarity to Ideal Solution) method is an ordering method that approximates the ideal solution. This method was first proposed by Hwang C., and Yoon K. in 1981 for solving program evaluation and selection in a multi-attribute decision-making process [46]. Considering the application of hospital alliance resource urgency evaluation, we improve the method as follows:

(1) Suppose the hospital alliance set $A=\{a_{1},a_{2},\cdots ,a_{m}\}$ contains m hospitals, and the urgency impact factors set $K=\left\{k_{1},k_{2},\cdots ,k_{n}\right\}$ contains n impact factors. The weight result is $\Omega =\left\{\omega_{1},\omega_{2},\cdots ,\omega_{n}\right\}$ and $\omega_{i}\geq 0,i=1,2,\cdots ,n$. Define the $x_{ij}$ member i value of the impact factor $k_{j}$, and the initial evaluation vertex X as Eq. (11) shows.


$$X=\left[\begin{array}{cccc} {}*20cx_{`11} & x_{`12} & \cdots & x_{`1n}\\ x_{`21} & x_{`22} & \cdots & x_{2m}\\ \vdots & \vdots & \ddots & \vdots \\ x_{`m1} & x_{`m2} & \cdots & x_{`mn} \end{array}\right],$$ (11)

(2) The vector normalization method is used to normalize the matrix. Let the normalized decision matrix $Z=\left\{z_{ij}\right\}$ be as Eq. (12) follows:


$$z_{ij}=\frac{x_{ij}}{\sqrt{\sum {\mathrm{\backslash nolimits}}_{i=1}^{m}x_{ij}^{2}}},i=1,2,\cdots ,m;j=1,2,\cdots ,n$$ (12)

(3) Design the weighted normative matrix $\overset{\wedge }{X}=\left\{\overset{\wedge }{x_{ij}}\right\}$, and the matrix is shown as follows. In the Eq. (13), $\overset{\wedge }{x_{ij}}=\omega_{j}z_{ij},i=1,2,\cdots ,m\;;j=1,2,\cdots ,n$.


$$\overset{\wedge }{X}=\left\{\begin{array}{cccc} {}*20c\omega_{1}z_{11} & \omega_{2}z_{12} & \cdots & \omega_{n}z_{1n}\\ \omega_{1}z_{21} & \omega_{2}z_{22} & \cdots & \omega_{n}z_{2n}\\ \vdots & \vdots & \ddots & \vdots \\ \omega_{1}z_{m1} & \omega_{2}z_{m2} & \cdots & \omega_{n}z_{mn} \end{array}\right\},$$ (13)

(4) Define the positive and negative ideal solutions

In the process of determining the positive and negative ideal solutions of the urgency influence factor, it is necessary to differentiate according to the reality of the influence factor. For impact factors of different natures, the direction of the positive and negative ideal solutions will be different. For example, for the negative impact factor belonging to the cost type, the positive ideal solution $x_{j}^{*}$ takes the minimum value of the attributes in the program to be evaluated, and the negative ideal solution $x_{j}^{0}$ takes the maximum value of the attribute j in the program to be evaluated; for the positive impact factor belonging to the benefit type, the positive ideal solution $x_{j}^{*}$ takes the maximum value of the attributes in the program to be evaluated, and the negative ideal solution $x_{j}^{0}$ takes the minimum value of the attribute j in the program to be evaluated, as shown in Eq. (14) and Eq. (15).

The positive ideal solution is as follows:


$$x_{j}^{*}=\left\{ \begin{array}{c} \underset{i\in m}{\text{max}}x_{ij}\;j\;\text{is}\;\text{cost}\;\text{type}\\ \underset{i\in m}{\text{min}}\;x_{ij}\;j\;\text{is}\;\text{revenue}\;\text{type} \end{array}\right.\;,\;j=1,\;2,\;\ldots ,\;n$$ (14)

The negative ideal solution is as follows:


$$x_{j}^{0}=\left\{ \begin{array}{c} \underset{i\in m}{\text{max}}x_{ij}\;j\;\text{is}\;\text{cost}\;\text{type}\\ \underset{i\in m}{\text{min}}\;x_{ij}\;j\;\text{is}\;\text{revenue}\;\text{type} \end{array}\right.\;,\;j=1,\;2,\;\ldots ,\;n$$ (15)

**Fig 6:**
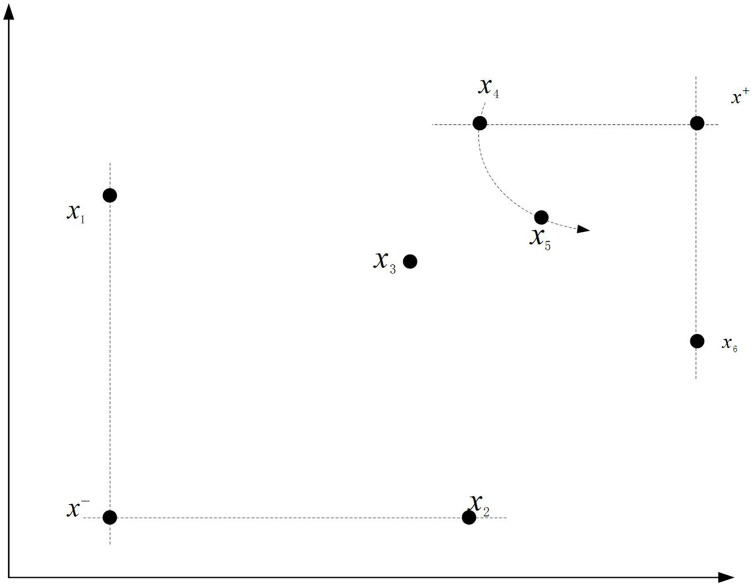
Graphical representation of positive and negative ideal solutions.

In the calculation process, the positive ideal solution $x^{*}$ is the virtual hospital that does not exist in the set of members of the hospital alliance, and each attribute value of the virtual hospital is the highest value of the urgency attribute; the negative ideal solution $x^{0}$ is the virtual hospital that does not exist in the members of the hospital alliance, and each attribute value of the virtual hospital is the lowest value of the urgency influence factor, and the construction of the positive and negative ideal solutions is shown in Fig 6. In the entropy-TOPSIS model, the positive and negative ideal solutions is very important, and the calculation of urgency values $C_{i}$ is closely related to the positive ideal solution $x^{*}$ and negative ideal solution $x^{0}$.

(5) Calculation of Euclidean distances between cluster member hospitals to positive and negative ideal solutions.

Euclidean Distance, a mathematical tool for evaluating distance proximity in graph theory, has the advantages of easy operation and strong interpretability. Introducing Euclidean Distance into program evaluation can effectively measure the gap between different attributes and positive and negative ideal values, which is calculated as follows.

The Euclidean distance to the positive ideal solution $x_{j}^{*}$ for the member hospital $x_{i}$ of the alliance is shown in Eq. (16).


$$d_{i}^{*}=\sqrt{\sum {\mathrm{\backslash nolimits}}_{j=1}^{n}{\left(x_{ij}-x_{j}^{*}\right)}^{2}},i=1,2,\cdots ,m$$ (16)

The Euclidean distance to the negative ideal solution $x_{j}^{0}$ for the member hospital $x_{i}$ of the alliance is shown in Eq. (17).


$$d_{i}^{0}=\sqrt{\sum {\mathrm{\backslash nolimits}}_{j=1}^{n}{\left(x_{ij}-x_{j}^{0}\right)}^{2}},i=1,2,\cdots ,m$$ (17)

(6) Calculating the numerical results of the urgency of hospitals that are members of the alliance.

Based on this calculation, the final quantified urgency value $C_{i}$ can be calculated as shown in Eq. (18). The structure of this equation also shows that the urgency value is in the interval [0,1] and that the larger the value, the higher the degree of urgency of the hospital’s supplies, and the smaller the value, the lower the degree of urgency of the supplies.


$$C_{i}^{*}=\frac{d_{i}^{0}}{d_{i}^{0}+d_{i}^{*}},i=1,2,\cdots ,m$$ (18)

**Step 1.** Import the initial data related to the calculation of the urgency of the medical association.

**Step 2.** Calculate the influence factor of medical cluster diagnosis and treatment urgency. Based on the complex network model, the matrix of the medical cluster diagnosis and treatment network was analyzed, and the node centrality, node strength, and node aggregation of each member hospital of the medical cluster were calculated.

**Step 3.** Calculate the epidemic urgency impact factor of the hospital alliance. Based on the SEIR infectious disease model, the outbreak dynamics in the jurisdiction of each node hospital of the hospital alliance were calculated, including the number of latent patients, the number of confirmed mild cases, and the number of confirmed severe cases.

**Step 4.** Based on expert experience and actual research visits, the degree of importance between different attributes is determined and an importance discrimination matrix is constructed.

**Step 5.** The weight matrix Ω is computed using the eigenvector method.

**Step 6.** For the weight vector consistency test, if the test passes then go to **Step 4**, if the test fails, then return to **Step 1**. What should be noticed is that the consistency test is primarily employed for the following purposes: to verify whether the derived weights align with domain experts’ judgments, to evaluate if the weight distribution reflects objective reality, and to detect potential computational anomalies or data irregularities.

**Step 7.** The entropy weight method is used to calculate the information entropy value and information entropy deviation value of each attribute and generate the entropy weight matrix of each attribute.

**Step 8.** The weight matrix of the eigenvector method is multiplied by the weight matrix of the entropy weight method, and then the processed matrix is summed, and the numerical share of each dimension is calculated so that the weight matrix satisfies the basic constraint of summing to one.

**Step 9.** The decision matrix for the multi-attribute problem is normalized by dividing each value within the matrix by the open value of the sum of the squares of the columns in which it is located.

**Step 10.** Integrate the canonical decision matrix with the results of **Step 5** weights to generate a weighted canonical matrix.

**Step 11.** Positive and negative ideal point setting. Based on the attribute characteristics of each demand point, it is clarified whether it is a benefit-type or cost-type characteristic, and the positive and negative ideal solutions are set accordingly.

**Step 12.** The Euclidean distance formula in multidimensional space was used to calculate the distance from each demand point to the positive and negative ideal points.

**Step 13.** Calculation of urgency results for the hospital alliance. The numerical results of material urgency for each member hospital of the hospital alliance were calculated by the entropy-TOPSIS comprehensive evaluation method based on the Euclidean distance of each hospital from the positive and negative ideal points.

## 5 Case study

### 5.1 Case background

The Omicron COVID-19 pandemic broke out in Beijing at the end of 2022, and major hospitals in the capital experienced varying degrees of shortage of medical supplies, with a huge gap between the supply and demand of medicines. The Haidian hospital alliance is one of the first comprehensive medical consortia in Haidian District, Beijing, and belongs to the Central and Western hospital alliance of Haidian District. The hospital alliance is led by Beijing Haidian Hospital, and the main members of the alliance consist of 12 healthcare organizations in the Haidian district, including first-level hospitals, second-level hospitals, and third-level hospitals. At the end of 2022, the new Crown Omicron variant strain was widespread in the Beijing area, and there was high pressure on the diagnosis and treatment of the member organizations of the Haidian hospital alliance. Haidian Hospital, as the lead unit of its hospital alliance, promotes hierarchical diagnosis and treatment, improving patient care efficiency and achieving strong results.

The members of the Haidian hospital alliance are shown in Table 2, in which Haidian Hospital is the lead hospital, and other medical institutions are members of the hospital alliance. Due to the lack of a quantified urgency evaluation method, it is difficult to identify the priority of support for the member hospitals. Meanwhile, in terms of the distribution of emergency medicines in the alliance, the Haidian Hospital alliance also lacks quantitative methods and models to support it, and in actual operation, it can only distribute the existing stock of medicines based on its historical experience, and then conduct the procurement and distribution of incremental medicines from outside of the medical institution based on its historical experience. The lack of science in the distribution of emergency medical supplies in the medical association has led to the irrational distribution of medicines in the Haidian hospital medical association, which affects the satisfaction level of the patients who go to the Haidian medical association to obtain medicines. Lack of sufficient medical resources could also lead to the rapid diffusion of this epidemic.

**Table 2:** Detailed information on hospital alliance.

ID	Name	Hospital level	Administrative district	Initial quantity
1	Haidian hospital	Third-level	Haidian	4200
2	Zhongguancun hospital	Second-level	Haidian	1821
3	Haidian district maternal and Child Health Hospital	Second-level	Haidian	1535
4	Haidian district emergency care center	Second-level	Haidian	500
5	Shuangyushu community health service center	Third-level	Haidian	980
6	Qinglongqiao community health service center	Third-level	Haidian	850
7	Haidianzhen community health service center	Third-level	Haidian	900
8	Zhongguancun community health service center	Third-level	Haidian	950
9	Renmin University of China health service center	Third-level	Haidian	400
10	Beijing institute of technology health service center	Third-level	Haidian	350
11	China meteorological administration hospital	Third-level	Haidian	720
12	Beijing wanshoukang hospital	Third-level	Haidian	600

Based on the medical expert’s discussion, the importance weight matrix Ω=[235123] is used. According to the weight matrix, the impact factor significance discrimination matrix is shown in Table 3. (①Medical network node centrality factor, ②Medical network node clustering factor, ③Medical network node shortage factor, ④Number of latency patients, ⑤Number of mild patients, ⑥ Number of critical patients).

**Table 3:** Impact factors significance discrimination matrix.

	①	②	③	④	⑤	⑥
①	1	–	–	–	–	–
②	3/2	1	–	–	–	–
③	5/2	5/3	1	–	–	–
④	1/2	1/3	1/5	1	–	–
⑤	1	2/3	2/5	2	1	–
⑥	3/2	1	3/5	3	3/2	1

The above data were sourced from the official websites of member institutions within the Beijing Haidian hospital alliance Network. In this network, nodes represent hospitals, and edges represent referral and resource allocation relationships between them. The parameters in the SEIR model were defined based on the classic SEIR theoretical framework. For the entropy weight calculation, this study adopts a combined qualitative and quantitative approach. Through expert interviews with hospital specialists, we assessed the relative importance of each weight before determining its values in the case study. It should be noted that the weight values used here serve only as a reference for this specific case. For different scenarios, the weight design can be dynamically adjusted according to the specific circumstances of public health emergencies.

### 5.2 Impact factor calculation

Based on Table 2 and Table 3, the patient number data of each member hospital of the hospital alliance can be calculated, and the results of the patient number of each member hospital of the hospital alliance of the Haidian Hospital can be obtained. Medical referral data and population data are in the S1 Data. In terms of referral of patients with minor illnesses, the ratio of referral from community hospitals to Haidian Hospital is 10%, and the ratio of referral to each of the two secondary hospitals is 20%; in terms of referral of patients with serious illnesses, the ratio of referral from community hospitals to Haidian hospital is 80%, and only to Haidian hospital. The final calculation results are shown in Table 4.

**Table 4:** The result of the medical treatment impact factor.

ID	Name	Node centrality	Node aggregation	Resource gap
1	Haidian hospital	0.122	0.618	0.300
2	Zhongguancun hospital	0.122	0.618	0.521
3	Haidian district maternal and Child Health Hospital	0.111	0.667	0.452
4	Haidian district emergency care center	0.122	0.618	0.706
5	Shuangyushu community health service center	0.089	0.786	0.694
6	Qinglongqiao community health service center	0.056	1.000	0.757
7	Haidianzhen community health service center	0.056	1.000	0.719
8	Zhongguancun community health service center	0.056	1.000	0.661
9	Renmin University of China health service center	0.056	1.000	0.875
10	Beijing institute of technology health service center	0.056	1.000	0.865
11	China meteorological administration hospital	0.056	1.000	0.520
12	Beijing wanshoukang hospital	0.100	0.722	0.727

### 5.3 Results

By inputting the six-dimensional factors into the entropy-TOPSIS model, the resource urgency of the Haidian hospital alliance can be calculated, as shown in Table 5, and the urgency values of each member unit are all in the [0,1] interval. The closer the value is to 1, the greater the urgency is represented.

**Table 5:** The calculation result of Haidian hospital alliance.

ID	Name	Urgency degree	Ranking
1	Haidian hospital	0.376	10
2	Zhongguancun hospital	0.378	9
3	Haidian district maternal and Child Health Hospital	0.327	11
4	Haidian district emergency care center	0.506	7
5	Shuangyushu community health service center	0.538	6
6	Qinglongqiao community health service center	0.543	5
7	Haidianzhen community health service center	0.579	3
8	Zhongguancun community health service center	0.485	8
9	Renmin University of China health service center	0.629	1
10	Beijing institute of technology health service center	0.605	2
11	China meteorological administration hospital	0.305	12
12	Beijing wanshoukang hospital	0.546	4

According to the above discussion, in public health emergencies, the material urgency level of community hospitals is significantly higher than that of large hospitals. In the above case study, the Renmin University of China Health Service Center has the highest material urgency level at 0.629, while Haidian Hospital, as a large tertiary hospital, has a material urgency level of only 0.376. What should be noticed is that existing empirical evidence has validated this conclusion. During a press conference addressing the Omicron outbreak, National Health Commission of the People’s Republic of China emphasized that small medical institutions in communities and rural areas have indeed faced significant material shortages and clinical pressure in practice. The Commission stressed the need to strengthen material support for these smaller facilities by implementing the hospital alliance [47].

### 5.4 Sensitive analysis experiment

#### (1) Critical patient’s upward referral parameter.

Considering that referral is an important feature of medical alliances, this study conducted a sensitivity analysis experiment on the referral parameters of critically ill patients, with a step size of 0.1, ranging from 0.1 to 0.5, to observe the material urgency level in hospitals. The sensitive results are shown as follows. From Table 6, it can be seen that as the rate of referral of critically ill patients to higher levels increases, the material urgency rank ordering of large hospitals (Haidian Hospital) increases, verifying the direct effect of critical care referral parameters on urgency ordering results.

**Table 6:** Sensitive analysis of critical patients’ upward referral for urgency sequence.

ID	Hospital name	Critically patients’ upward referral ratio				
		10%	20%	30%	40%	50%
1	Haidian hospital	9	8	1	1	1
2	Zhongguancun hospital	11	11	11	11	11
3	Haidian district maternal and Child Health Hospital	12	12	12	12	12
4	Haidian district emergency care center	7	7	8	8	6
5	Shuangyushu community health service center	8	9	9	9	9
6	Qinglongqiao community health service center	5	4	5	5	4
7	Haidianzhen community health service center	2	2	3	3	5
8	Zhongguancun community health service center	4	5	7	7	7
9	Renmin University of China health service center	3	3	4	2	2
10	Beijing institute of technology health service center	6	6	6	4	3
11	China meteorological administration hospital	10	10	10	10	10
12	Beijing wanshoukang hospital	1	1	2	6	8

#### (2) Weight importance parameters.

In processing data using the entropy-TOPSIS model, variations in the multiple dimensions’ weight parameters can also have an impact on the results. Therefore, in this paper, a sensitivity analysis of the importance weight matrix will be performed to test the impact of the results under different importance weight matrices. In this section, five sets of experiments were conducted, and the importance matrix of weights is as follows:$\Omega_{1}=\left[111111\right]$,$\Omega_{2}=\left[222111\right]$,$\Omega_{3}=\left[333111\right]$,$\Omega_{4}=\left[444111\right]$,$\Omega_{5}=\left[555111\right]$. The result is shown in Table 7. As can be seen from the table, the urgency of supplies in second-level hospitals that receive a higher number of patients increases significantly with the increase in the importance weights of the variables related to diagnosis and treatment, thus verifying that changes in the parameters of the importance matrix have an impact on the results.

**Table 7:** Sensitive analysis of importance matrix for urgency sequence.

ID	Hospital name	Weight importance matrix				
		$\Omega_{1}$	$\Omega_{2}$	$\Omega_{3}$	$\Omega_{4}$	$\Omega_{5}$
1	Haidian hospital	1	3	9	9	9
2	Zhongguancun hospital	10	10	10	10	10
3	Haidian district maternal and Child Health Hospital	11	12	12	12	12
4	Haidian district emergency care center	8	5	2	2	1
5	Shuangyushu community health service center	5	6	6	5	5
6	Qinglongqiao community health service center	7	8	5	4	4
7	Haidianzhen community health service center	2	2	4	6	6
8	Zhongguancun community health service center	9	9	8	7	7
9	Renmin University of China health service center	3	1	1	1	2
10	Beijing institute of technology health service center	6	4	3	3	3
11	China meteorological administration hospital	12	11	11	11	11
12	Beijing wanshoukang hospital	4	7	7	8	8

The reasons for the aforementioned findings are threefold. First, small medical institutions have limited medical supply reserves, making their stockpiles insufficient when facing a large number of infected residents seeking treatment. Second, large hospitals maintain larger routine inventories and have well-established daily replenishment protocols for emergency supplies, making them relatively better stocked compared to smaller facilities. Third, due to the cyclical nature of infectious disease transmission, residents in the early stages often exhibit milder symptoms and tend to seek medication or care at nearby community or rural clinics, which would alleviate some of the patient load in larger hospitals.

## 6. Conclusion, recommendations and future works

### 6.1 Conclusion

This paper investigates the characteristics of material urgency quantification within hospital alliances, clarifies the heterogeneity of material urgency among member hospitals, and designs a methodology for quantifying material urgency in such alliances. Based on the above discussion and numerical analysis, we strongly draw the following conclusions.

(1) This paper proposed a novel comprehensive urgency evaluation model for emergency supplies in a hospital alliance and effectively verified the effectiveness and feasibility of this method with a reliable computer simulation approach. In the public health emergency scenario, the referral mechanism of the hospital alliance helps to optimize the coordination of resources and alleviate the degree of urgency of supplies for some members of the hospital alliance, which in turn promotes the optimal allocation of resources. Meanwhile, this paper also first unveiled that the key factors affecting the urgency of the hospital alliance are well-designed, which was verified by the case study.
(2) This paper strongly unveiled that small community hospitals face greater material shortages than large hospitals during public health events. Specifically, the urgency degree of Haidian hospital is 0.376, but the Renmin University of China Health Service Center’s is 0.628, which is almost twice as much as Haidian hospital. Consequently, during public health emergencies, community hospitals assume critical roles and perform essential functions in the integrated healthcare delivery system. The findings are not only applicable to medical supply management in China’s healthcare alliances but also could be extended to the urgent assessment and management of scarce medical supplies in other countries or regions.
(3) This paper strongly found that the referral ratio parameters and indicator weight parameters of hospital alliance have high parameter sensitivity to results and need to be set with caution in practical application. As the proportion of referred patients increased from 10% to 50%, the urgency of supplies for the Haidian Hospital alliance also changed. Specifically, Haidian Hospital’s urgency ranking rose from 9th to 1st, while Beijing wanshoukang Hospital fell from 1st to 8th. Additionally, in five sets of sensitivity experiments involving weighting parameters, as the weighting of medical network-related parameters increased, Haidian Hospital’s urgency ranking decreased from 1st to 9th.

### 6.2 Recommendations

Based on the simulation analysis and case study in this paper, the following three management recommendations are proposed to support hospital alliance administrators in allocating medical supplies during public health emergencies.

(1) **The managers of the hospital alliance and local government must accelerate efforts to attract hospitals to join the** hospital alliance **network.** If there is a lack of coordination in the allocation of medical supplies between hospitals, it will negatively impact the overall regional healthcare delivery and hinder residents’ access to medical services. Therefore, when responding to public health emergencies, it is recommended that medical institutions join the alliance as soon as possible and adopt the mechanism of alliance’s operation mechanism for the scientific distribution of emergency supplies.
(2) **The managers of the hospital alliance should pay more attention to the shortage of supplies in community hospitals and small hospitals.** The community hospitals (small-scale hospitals) within the alliance have faced heightened patient flow pressure, exacerbating the gap between medical resource supply and demand. Therefore, during public health emergencies, it is recommended that administrators of hospital alliances should carry out a scientific assessment to evaluate the urgency of the specific resources from a systematic perspective.
(3) **The managers of the hospital alliance must conduct dynamic assessments of the urgency of supplies, as patient referrals will update the urgency of supplies within the hospital alliance.** The patient transfer mechanism embedded in the hospital alliance would lead to many patients turning to small-scale hospitals for medical treatment and then transferring to higher-level hospitals. Therefore, hospital alliance management departments need to carefully set referral ratios when conducting referral work and dynamically update and adjust the severity of material shortages within medical consortia to achieve optimal resource allocation.

### 6.3 Future works

Throughout history, the management of public health emergencies has evolved alongside human civilization and is closely linked to the survival of human populations. Therefore, research on this issue will be an ongoing, dynamic process. This study focuses on the issue of resource allocation during public health emergencies and proposes a model for assessing resource urgency. However, given the limitations of the times and the rapid advancements in technology, future emergency resource management will require research that better aligns with the characteristics of the era. The following sections offer some insights into future research directions, with the hope of inspiring readers.

Regarding future research directions, we believe emerging technologies and organizational innovations will reshape hospital alliance operations, warranting in-depth exploration. With the digitalization of the hospital alliance, the resource evaluation framework and methodology should also be changed to meet the coming digital medical era. For example, the boom of LLM (Large language model) would promote the management level of medical storage and provide a more specific value for the urgency evaluation model. Meanwhile, the data-driven method could also be developed and embedded in this proposed methodology to make the parameter’s value more scientific. In this way, we could improve the proposed urgency evaluation model in the future to meet the technological changes in the hospital alliance. We hope that this study will provide support for the management of core medical supplies in global healthcare systems in response to public health emergencies, thereby promoting the advancement of global emergency healthcare.

## Supporting information

S1 Data The supporting file “Data on patient referrals to Beijing Haidian Hospital” is the details of hospital referral numbers. (DOCX)
