# Expression of Concern: Computational modeling and analysis of medical resource shortages in hospital alliance: A simulation-driven approach

**DOI:** 10.1371/journal.pone.0351179

**Published:** 2026-06-09

**Authors:** The PLOS One Editors

After this article [1] was published, the following concerns were noted:

- The article uses terminology that differs from standards in the field (e.g. ‘new crown epidemic’ instead of ‘Corona pandemic’).
- The articles cited as Reference 32 and 33 were retracted before [1] was published.
- The articles cited as Reference 41 and 45 do not appear to support the statements for which they were cited.

In light of the cumulative issues, the *PLOS One* Editors issue this Expression of Concern. Readers are advised to interpret the article [1] with caution.

## References

[1] Gan Z, Fu Z, Dong P, Ju Y, Shen Y. Computational modeling and analysis of medical resource shortages in hospital alliance: A simulation-driven approach. PLoS One. 2025;20(8):e0330871. doi: 10.1371/journal.pone.0330871 40857292 PMC12380337

