# Supplementary material for: Computational modeling and analysis of medical resource shortages in hospital alliance: A simulation-driven approach
Source: PLoS One. 2025 Aug 26;20(8):e0330871. doi: 10.1371/journal.pone.0330871 (PMC12380337; doi:10.1371/journal.pone.0330871)
Supplement: S1 Data — (DOCX) [file pone.0330871.s001.docx]

Appendix A Data on patient referrals to Beijing Haidian Hospital's medical cluster

Table A1 Matrix for two-way referral of patients to the medical alliance

|  | Haidian hospital | Zhongguancun hospital | Haidian district maternal and Child Health Hospital | Haidian district emergency care center |
| --- | --- | --- | --- | --- |
| Haidian hospital | 0 | — | — | — |
| Zhongguancun hospital | 4200 | 0 | — | — |
| Haidian district maternal and Child Health Hospital | 2522 | 690 | 0 | — |
| Haidian district emergency care center | 3654 | 1230 | 1090 | 0 |
| Shuangyushu community health service center | 1250 | 320 | 800 | 820 |
| Qinglongqiao community health service center | 1103 | 635 | 650 | 370 |
| Haidianzhen community health service center | 960 | 235 | 360 | 560 |
| Zhongguancun community health service center | 1562 | 569 | 560 | 210 |
| Renmin University of China health service center | 2103 | 236 | 90 | 160 |
| Beijing institute of technology health service center | 1356 | 136 | 50 | 230 |
| China meteorological administration hospital | 890 | 268 | 0 | 100 |
| Beijing wanshoukang hospital | 360 | 120 | 203 | 36 |

Table A1(continuation sheet) Matrix for two-way referral of patients to the medical alliance

|  | Shuangyushu community health service center | Qinglongqiao community health service center | Haidianzhen community health service center | Zhongguancun community health service center |
| --- | --- | --- | --- | --- |
| Haidian hospital | — | — | — | — |
| Zhongguancun hospital | — | — | — | — |
| Haidian district maternal and Child Health Hospital | — | — | — | — |
| Haidian district emergency care center | — | — | — | — |
| Shuangyushu community health service center | 0 | — | — | — |
| Qinglongqiao community health service center | 0 | 0 | — | — |
| Haidianzhen community health service center | 0 | 0 | 0 | — |
| Zhongguancun community health service center | 0 | 0 | 0 | 0 |
| Renmin University of China health service center | 450 | 0 | 0 | 0 |
| Beijing institute of technology health service center | 650 | 0 | 0 | 0 |
| China meteorological administration hospital | 130 | 0 | 0 | 0 |
| Beijing wanshoukang hospital | 65 | 98 | 100 | 69 |

Table A1(continuation sheet) Matrix for two-way referral of patients to the medical alliance

|  | Renmin University of China health service center | Beijing institute of technology health service center | China meteorological administration hospital | Beijing wanshoukang hospital |
| --- | --- | --- | --- | --- |
| Haidian hospital | — | — | — | — |
| Zhongguancun hospital | — | — | — | — |
| Haidian district maternal and Child Health Hospital | — | — | — | — |
| Haidian district emergency care center | — | — | — | — |
| Shuangyushu community health service center | — | — | — | — |
| Qinglongqiao community health service center | — | — | — | — |
| Haidianzhen community health service center | — | — | — | — |
| Zhongguancun community health service center | — | — | — | — |
| Renmin University of China health service center | 0 | — | — | — |
| Beijing institute of technology health service center | 0 | 0 | — | — |
| China meteorological administration hospital | 0 | 0 | 0 | — |
| Beijing wanshoukang hospital | 0 | 0 | 263 | 0 |

Appendix B Population data of Beijing Haidian Hospital's medical alliance

Table B1 Data on the population covered by each hospital district of the medical association

|  | Population covered by hospital districts | Ageing proportion of hospital district population |
| --- | --- | --- |
| Haidian hospital | 400000 | 25% |
| Zhongguancun hospital | 300000 | 18% |
| Haidian district maternal and Child Health Hospital | 250000 | 12% |
| Haidian district emergency care center | 100000 | 18% |
| Shuangyushu community health service center | 140000 | 12% |
| Qinglongqiao community health service center | 180000 | 20% |
| Haidianzhen community health service center | 130000 | 20% |
| Zhongguancun community health service center | 150000 | 22% |
| Renmin University of China health service center | 120000 | 15% |
| Beijing institute of technology health service center | 100000 | 15% |
| China meteorological administration hospital | 40000 | 25% |
| Beijing wanshoukang hospital | 100000 | 35% |
